# Hydrological properties predict the composition of microbial communities cycling methane and nitrogen in rivers

**DOI:** 10.1038/s43705-022-00087-7

**Published:** 2022-01-21

**Authors:** Dave R. Clark, Boyd A. McKew, Andrew Binley, Catherine M. Heppell, Corinne Whitby, Mark Trimmer

**Affiliations:** 1 School of Life Sciences, University of Essex, Wivenhoe Park, Colchester, Essex CO4 3SQ UK; 2 Institute for Analytics and Data Science, University of Essex, Wivenhoe Park, Colchester, Essex CO4 3SQ UK; 3 Lancaster Environment Centre, Lancaster University, Lancaster, LA1 4YQ UK; 4 School of Geography, Queen Mary University of London, Mile End Road, London, E1 4NS UK; 5 School of Biological and Behavioural Sciences, Queen Mary University of London, Mile End Road, London, E1 4NS UK

**Keywords:** Microbial ecology, Molecular ecology, Environmental microbiology

## Abstract

Sediment microbial communities drive the biogeochemical cycles that make rivers globally important sources and sinks of carbon (C) and nitrogen (N). The structure of these communities is strongly determined by the local physico-chemical environment. However, we currently lack an understanding of the factors that determine microbial community structures at the catchment scale. Here, we show that the contribution of groundwater to total river flow (quantified as base flow index; BFI) predicts the structure and diversity of the different microbial functional groups that cycle N and C across nine UK rivers, spanning a geological BFI gradient from 0.23 (clay sediment) to 0.95 (chalk gravel sediment). Furthermore, the GC-content (percentage of guanine-cytosine bases in a DNA sequence) and codon-usage bias of ammonia monooxygenase DNA sequences, and the hydrophobicity and net-charge of the corresponding amino acid sequences, were all strongly correlated with BFI, likely reflecting physiological adaptations to different riverbed sediment structure along the BFI gradient. Our results offer an opportunity to overcome the “paradox of scales” that has seen microbial ecologists focus on small- rather than large-scale environmental variables, enabling us to scale-up our understanding of microbial biogeochemistry to the catchment and beyond.

## Introduction

Rivers play a crucial role in the biogeochemical cycles of key macronutrients such as nitrogen (N) and carbon (C). Not only do rivers transport 0.4 Pg of C [1] and 61.5 Tg of N [2] per year to the sea, but they are increasingly recognised as key players in global biogeochemical cycles. Less than half of the C pool transported by rivers reaches the coast [3] whilst around 40% of terrestrial N-runoff is converted to inert atmospheric N_2_ gas within rivers [4], highlighting the ability of rivers to attenuate and transform macronutrients.

Within rivers, sediment microbial communities are the major drivers of C- and N-cycles, and thus control the abundance and forms of these nutrients. Consequently, understanding the environmental and ecological drivers of these functionally important microbial communities has remained a key priority in advancing our knowledge of riverine biogeochemistry [5–9]. To date, most microbiological research in rivers and other ecosystems has focussed on the small-scale physico-chemical environment. Consequently, extrapolating ecological patterns to understand microbial biogeochemistry at spatial scales beyond single rivers has remained challenging.

The ability of landscape-scale geodiversity variables—the diversity of geology, landforms and abiotic processes [10]—to explain microbial community dynamics offers a viable, but understudied, route to scale-up microbial ecological research [11]. Along the river continuum, from headwaters to estuaries, shifts in hydraulic conditions alter microbial functional profiles [12, 13], but the landscape-scale variables that explain differences in community composition between similar order streams and rivers remain elusive.

Base flow is the contribution of flow to a river from delayed groundwater pathways. The base flow index (BFI) of a river is the ratio of flow from base flow to total river flow and ranges from 0 (no contribution of delayed groundwater flow) to 1 (river totally fed from delayed groundwater) [14] and, as such, reflects the permeability of the catchment. Catchment permeability is largely determined by underlying geology, but also depends on surrounding land use and soil type [15–17]. Similar factors can also influence the riverbed physicochemistry at local scales. Our previous work in the Hampshire Avon catchment (UK) has shown BFI to be highly correlated with various physico-chemical variables including pore-water oxygen concentration, sediment particle size, dissolved organic C, and pH [8, 17, 18]. BFI therefore integrates up-stream catchment permeability and thus offers a potential path to upscale our understanding of the microbial communities driving fluvial biogeochemistry.

Here, we test the ability of BFI to predict the diversity, composition, and abundance of six C- and N-cycling microbial functional groups across a BFI gradient, spanning impermeable clay sediments (BFI 0.234–0.635), moderately permeable greensand sediments (BFI 0.695–0.868) and highly permeable chalk sediments (BFI 0.838–0.953). We demonstrate that the BFI alone explains up to 35% of the variation in community diversity, and 58% of the variation in community composition. Furthermore, we show that the predictive power of BFI extends beyond broad community-level properties to various enzymatic characteristics of biogeochemically significant functional genes, highlighting BFI as a potential route to studying fluvial biogeochemistry at the catchment-scale.

## Methods

### Field sampling

**Fig. 1:**
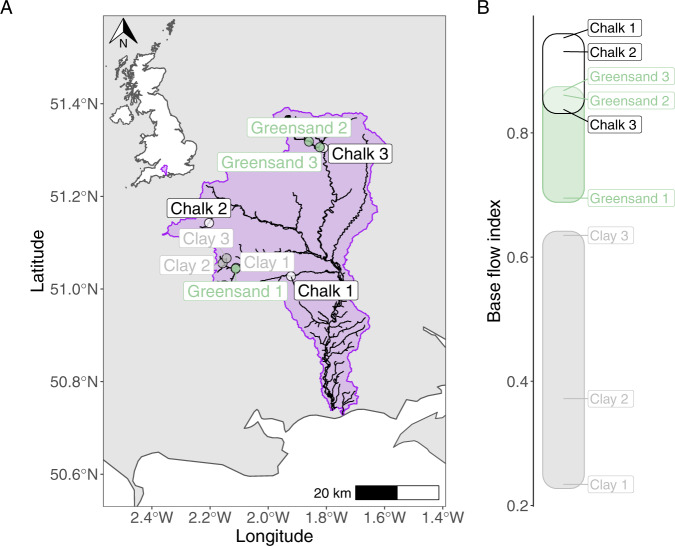
Sampling locations within the Hampshire Avon river catchment. A map of sampled rivers (**A**) with the inset map (top left) highlighting the position of the Hampshire Avon catchment within the United Kingdom. Spatial data used in this map were obtained from the Environment Agency Catchment Data Explorer (https://environment.data.gov.uk/catchment-planning). **B** The base flow index (BFI) of the sampled rivers, which is the ratio of flow from base flow to total river flow and ranges from 0 (no contribution of delayed groundwater flow) to 1 (river totally fed from delayed groundwater).

Sampling was conducted during February (winter), April (spring), August (summer) and November (autumn) in 2013 at nine river sites within the Hampshire Avon catchment (southern England), as described in [8]. Three sites were selected from each of three geologically contrasting sub-catchments (clay, Greensand and Chalk, Fig. 1) that differ in permeability, thus maximising the range of base flow conditions observed. The BFI for each site was calculated previously from a 2-year discharge dataset [17], using the hydrograph separation procedure [14]. At each site, three replicate sediment cores (9 cm diameter) were taken from the middle of the river channel, representing 0–5 cm depth, avoiding areas with substantial macrophyte growth, as described by [8]. A smaller single sediment subsample was taken from each core, homogenised and cryogenically preserved in a vapour shipper, before being stored at −20 °C prior to molecular analyses. Our total dataset therefore consisted of 108 samples (4 seasons × 9 sites × 3 replicate sediment samples = 108 samples). Pore water chemistry was measured as described previously [8].

### Molecular analyses

DNA was extracted from 0.25 g of frozen sediment using a PowerSoil DNA isolation kit (MO BIO Laboratories), following the manufacturer’s protocol. N (*amoA, nirS, hzo*) and C (*mcrA*) cycle functional gene abundances and bacterial 16S rRNA gene abundances were quantified by qPCR using a SensiFAST SYBR No-ROX kit (Bioline) on a CFX96 Touch Real-Time PCR Detection system (BioRad), using gene-specific primer sets (Table S1). Briefly, gene abundances were quantified against an internal standard calibration curve using DNA standards of each target gene from 10^2^ to 10^7^ copies in 20 μl reactions, containing 200 nM of primers and 1 μl of DNA template. Cycle conditions for all genes were 95 °C for 3 mins followed by 40 cycles at 95 °C for 10 s then 60 °C for 30 s. Specificity of qPCR assays were confirmed via melt curve analysis. All qPCR amplifications were performed in triplicate and averaged (arithmetic mean) prior to downstream statistical analysis.

Amplicon sequencing of N (*amoA, nirS, hzo*) and C (*mcrA, pmoA*) cycle genes and of phylogenetic marker genes (archaeal and bacterial 16S rRNA) was performed for samples collected in February and August (*n* = 54 samples). All genes were analysed via locus-specific primer sets to exclude non-specific amplification (e.g. between *pmoA* and *amoA* genes). Each primer was flanked by an Illumina-specific overhang sequence that enables multiplexing indices and MiSeq flow cell binding sequences to be attached downstream (see Table S1 for further details). PCR conditions for ammonia oxidising archaea (AOA) and bacteria (AOB) *amoA* genes and anaerobic ammonium oxidising (anammox) bacterial *hzo* genes followed those described previously [8, 19]. PCR conditions for other genes replicated those in Clark et al. [19] either exactly (archaeal 16S rRNA, bacterial 16S rRNA), or with modified annealing temperatures (*nirS* = 57 °C, *mcrA* = 60 °C, *pmoA* = 56 °C). Preparation of sequencing libraries broadly followed those described by Illumina (https://support.illumina.com/downloads/16s_metagenomic_sequencing_library_preparation.html). Amplicons were initially bead-purified using Agencourt AMPure XP beads (Beckman Coulter Ltd), before a short eight-cycle PCR to attach sample-specific Nextera XT indices (Illumina). Indexed libraries were then bead purified again prior to quantification. Libraries were quantified using a Quant-iT PicoGreen dsDNA assay kit (Invitrogen) on a NanoDrop 3300 (Thermo Fisher), before being pooled in equimolar ratios. The quality and concentration of multiplexed libraries were verified using a DNA 1000 kit on an Agilent 2100 Bioanalyzer. Sequencing was conducted on an Illumina MiSeq using a V3 MiSeq Reagent kit (2 × 300 bp; Illumina) at the Earlham Institute (Norwich, UK).

### Bioinformatic analyses

For all functional genes, we used cutadapt (v 2.4 [20]) to remove primer sequences, using a minimum overlap 2–3 bp shorter than the primer length, and demultiplex libraries by primer sequence. Sequences were then quality-controlled using fastp (v 0.20.0 [21]) with the following criteria; minimum Phred score 20, maximum 20% of bases below min phred score, minimum length after trimming of 150 bp, minimum of 30% complexity in sequence, and a sliding window moving from 5′ to 3′. For genes where the amplicon length was short enough (all genes except archaeal *amoA*), we also used fastp to overlap paired-end sequences, allowing error correction in the overlap region, and specifying a minimum overlap of 10 bp. Errors were corrected using the BayesHammer algorithm with default settings, implemented in SPAdes (v 3.13.0 [22, 23]). Custom Linux shell scripts were then used to inspect sequence length distributions and to filter overly long or short sequences that could represent PCR artefacts or poor quality overlaps. The following length (*ɭ*) thresholds were used to filter sequences, archaeal *amoA; ɭ* ≥ 280 bp, bacterial *amoA; ɭ* ≥448 bp, *hzo*; 160 bp ≤ *ɭ* ≤ 190 bp, *nirS;* 360 bp ≤ *ɭ* ≤390 bp, *mcrA; ɭ* ≥ 415 bp, *pmoA; ɭ* ≥ 460 bp. Linux shell shell scripts were then used again to inspect the library size of each sample. Samples were discarded if they had fewer sequences than the following thresholds: archaeal *amoA;* 1800 sequences, bacterial *amoA;* 2600 sequences, *hzo*; 17,000 sequences, *nirS;* 3900 sequences, *mcrA;* 4900, *pmoA;* 3000, in order to ensure that remaining samples retained an adequate number of sequences after normalisation (details of final sample sizes are presented in Table S2). Phylogenetic marker gene (bacterial and archaeal 16S rRNA) libraries were analysed as described in [19] following protocols detailed in [24].

After filtering small library sizes from the dataset the remaining sequences were pooled. To correct any frameshift errors and remove any non locus-specific sequences from the functional gene datasets, we used a local installation of the FrameBot tool [25], which aligns and compares functional gene translated protein sequences to those in a database. For each gene, we assembled a custom database by downloading locus-specific protein sequences from the FunGene database [26] with a Hidden Markov Model coverage ≥90% and then de-replicated sequences using USEARCH (v 11.0667 [27]). For archaeal AmoA, the resulting database was too large to use with FrameBot (>18,000 sequences), so we randomly subsampled this database to 2000 sequences. The number of protein sequences in the de-replicated databases for other functional genes were as follows; bacterial AmoA = 306, anammox HZO = 2883, NirS = 197, McrA = 209, PmoA = 369. FrameBot was run using a minimum amino acid identity of 50% to remove non locus-specific sequences. The resulting sequences were then analysed at the amino acid level and nucleotide level. Firstly, to examine shifts in the putative functional composition of microbial communities, corrected protein sequences were dereplicated by sample using USEARCH, and used to create an amino acid variant (AAV) matrix. Here, an AAV represents a cluster of nucleotide sequences (with no fixed similarity threshold) that share an identical protein sequence, and thus any variation in nucleotide sequences within an AAV is silent. Secondly, a more traditional approach was used to examine shifts in community structure based on nucleotide sequences. Here, frameshift corrected nucleotide sequences output by FrameBot were clustered into operational taxonomic units (OTUs) at a 97% similarity level, using VSEARCH (v 2.10.2 [28]). Herein, we focus on the analysis of AAVs, but present results from OTU analyses for comparison in the supplementary materials.

### Statistical analyses

AAV and OTU tables were imported into R (v 3.6.2, [29]) and any AAVs or OTUs that occurred in only one sample were discarded prior to further analyses. OTU and AAV tables were rarefied independently to the minimum sample size of each table prior to statistical analyses (Methods and Table S2) in order to normalise variance due to unequal library sizes in an ecologically meaningful manner [30, 31]. To investigate the role of BFI on the β-diversity of AAV and OTU communities, pairwise community dissimilarity was quantified as Sørensen dissimilarity. To test the relationship between BFI and community turnover, we parameterised negative exponential functions using a generalised linear modelling (GLM) approach [32]. Goodness of fit was quantified as a pseudo-*R2*, defined as the reduction in deviance compared to a null model. The statistical significance of these relationships was quantified by a bootstrapping procedure using 1000 permutations. For each functional group, relationships to BFI were compared between AAV and OTU datasets by bootstrapping coefficients 1000 times. α-diversity was measured as either AAV or OTU richness, and was modelled as a function of BFI and geology using negative binomial GLMs. Adjusted *D*^*2*^ was calculated to quantify the explained deviance of a model. In addition, the qPCR-based abundance of AOA, AOB, and anaerobic ammonia-oxidisers as a proportion of the total ammonia-oxidising community (AOA + AOB + anammox), and of other functional groups as a proportion of the total bacterial community (16S rRNA gene copies) were analysed using β-GLMs. Coefficients are presented on the odds-scale.

To test the hypothesis that shifts in AAV composition were related to differences in protein hydrophobicity selected for by differences in base flow regimes, we calculated the Kyte-Doolittle hydrophobicity index for each AAV, and used this to calculate an average hydrophobicity index for each sample, weighted by the abundance of each AAV in each community. We then tested for any relationship between the average hydrophobicity and BFI or geology using linear regression. Similarly, we also calculated the net charge for each protein sequence, using the Lehninger pKa scale and assuming an intracellular pH of 7 for charge calculations.

The following R packages were necessary to conduct our analyses: vegan [33], betapart [34], MASS [35], Peptides [36], datatable [37], and ggplot2 [38]. Our catchment map (Fig. 1) was constructed using data available from the Environment Agency (UK) Catchment Data Explorer portal [39] and manipulated using the sf package in R [40]. All R/Linux shell scripts and data necessary to recreate our analyses are available in the Figshare repository under the 10.6084/m9.figshare.c.5404437. Raw sequence data are available in the NCBI sequence read archive under accession number PRJNA723875.

## Results and discussion

### Relationships between microbial diversity and base flow index

The number of reads obtained per sample and total number of OTUs obtained after rarefaction for each gene dataset are summarised in Table S2. According to taxonomic analyses of our 16S rRNA gene dataset, archaeal communities in our river sediment samples consisted largely of OTUs assigned to the Woesarchaeota (20.8% of OTUs and 24.7% of reads) and Methanomicrobia (16.9% of OTUs and 31.8% of reads). Of the functional groups analysed here, ten OTUs were assigned to AOA, *Nitrososphaera* (*n* = 8) and *Nitrosopumilus* (*n* = 2), that together formed 4.8% of all archaeal 16S rRNA reads. A total of 137 OTUs were assigned to orders of methanogenic archaea, with 15.3% and 16% of archaeal reads assigned to the orders Methanomicrobiales and Methanosarcinales, respectively, with other methanogen orders constituting a further 6.7% of reads.

Bacterial communities were more diverse and OTUs assigned to taxa within the functional groups analysed here formed a relatively small proportion of our bacterial 16S rRNA gene dataset. Ammonia oxidising bacteria were represented by only five OTUs (all assigned to *Nitrosospira*) that together constituted 0.02% of the total bacterial community across our sediments. A further 84 OTUs were assigned to methanotrophic genera, and these OTUs contributed a total of 0.88% of all bacterial 16S rRNA sequences. These were *Methylobacter* (30 OTUs, 0.7% of bacterial sequences), *Methylophilus* (15 OTUs, 0.1% of bacterial sequences), *Methylosoma* (7 OTUs, 0.004% of bacterial sequences), *Methylomonas* and *Methylotenera* (6 OTUs each, 0.02 and 0.002% of bacterial sequences, respectively), and *Methylosarcina* (5 OTUs, 0.002% of bacterial sequences), with a further eight genera represented by a total of 15 OTUs. As reported previously, no OTUs were assigned to known anammox genera, which were likely below the limit of detection in our study [8].

**Fig. 2:**
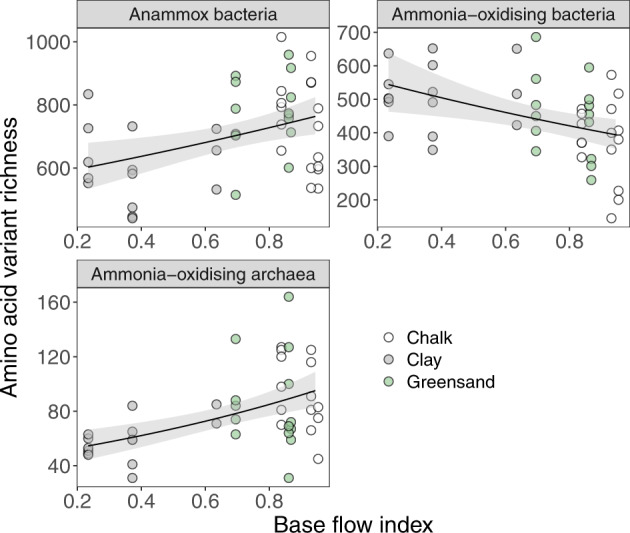
Relationships between base flow index (BFI) and the diversity of ammonia-oxidising microbial communities. The richness of amino acid variants (AAV) for anammox bacteria, ammonia oxidising bacteria and ammonia-oxidising archaea in relation to river BFI. Anammox bacterial richness was assessed using the hydrazine oxidoreductase (*hzo*) gene, whereas richness of aerobic ammonia-oxidising bacteria and archaea was quantified using the ammonia monooxygenase α-subunit (*amoA*) gene. Solid lines show statistically significant relationships (*P* < 0.05) and grey ribbons show 95% prediction intervals.

The OTU richness of archaeal communities (based on 16S rRNA amplicons) was negatively, albeit weakly, related to BFI (coef = 0.52, *z* = −2.95, adj-D^2^ = 0.12, *P* < 0.01), whereas bacterial OTU richness was not significantly related to BFI (coef = 0.88, *z* = −1.53, adj-D^2^ = 0.003, *P* = 0.13). The richness of both aerobic ammonia-oxidising bacteria and anammox bacteria changed along the BFI gradient, positively for anammox and negatively for AOB (Fig. 2 and Table S3). Ammonia oxidising archaea (AOA) increased in AAV richness in high BFI rivers, but not at the OTU level (Fig. 2). No relationships were observed between BFI and AAV or OTU richness for nitrite-reducing, methanotrophic, or methanogenic communities (Fig. S1). Temporal shifts in richness between summer and winter were rarely significant for any of the genes analysed (Fig. S1; Table S3).

**Fig. 3:**
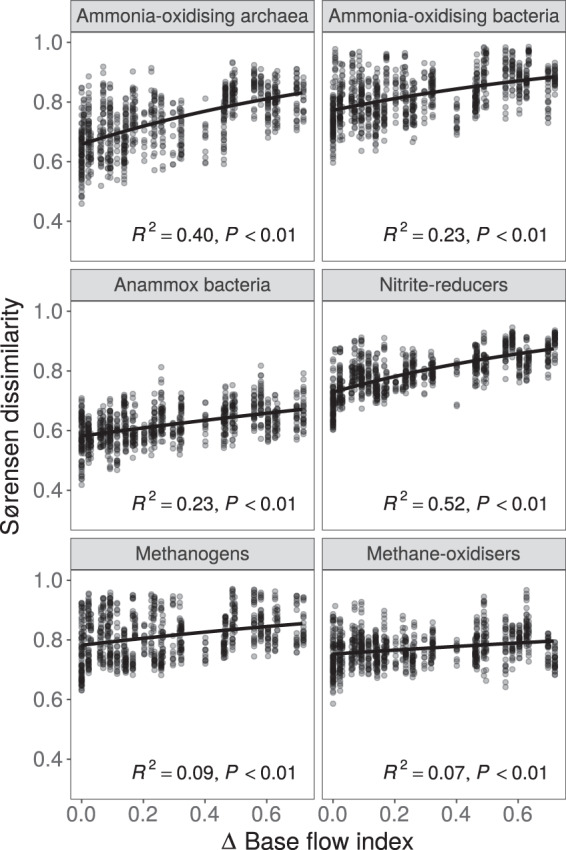
Relationships between base flow index (BFI) and microbial community dissimilarity. Relationships between pairwise differences in BFI and Sørensen dissimilarity for amino acid variant datasets. Sørensen dissimilarity values close to 1 indicate fewer shared species between communities. Lines show fit of negative exponential models and points are transparent to show their density. *R*^*2*^ and *P* values for each model are based on 1000 bootstraps.

Communities from rivers with similar BFI were compositionally more similar to each other than those from contrasting BFI rivers (Fig. 3 and S2). BFI explained the most variance in community composition for ammonia-oxidising archaea (AAV *R*^*2*^ = 0.40, *P* < 0.001; OTU *R*^*2*^ = 0.52, *P* < 0.001) and nitrite-reducing communities (AAV *R*^*2*^ = 0.52, *P* < 0.001; OTU *R*^*2*^ = 0.58, *P* < 0.001). In contrast, BFI had a less explanatory power for the composition of methane-oxidising and methanogenic communities (Fig. 3), and for the overall composition of the archaeal and bacterial communities.

Contrasting levels of phylogenetic diversity, encapsulated within the functional groups analysed here, could partly explain the different strengths of relationships between BFI and community composition due to phylogenetic niche conservatism. Aerobic and anaerobic ammonia oxidisers comprise relatively narrow phylogenetic diversity [41, 42] compared to methane-oxidisers and nitrite-reducers which are spread across several taxonomic classes [43, 44]. The strong relationship observed between BFI and nitrite-reducing community composition is therefore unexpected, as this group contains phylogenetically diverse species that, together, likely have a broad niche width. Potentially, the nitrite-reducers detected in our sediments represent only a limited subset of the phylogenetic diversity encompassed within this group, albeit some of which may overlap with taxa found in the other functional groups studied here due to *nir* genes being found in genomes from across the bacterial tree of life.

Shifts in community composition along the BFI gradient can be driven by turnover (the replacement of phylotypes along the gradient), or nestedness (the subsetting of communities along the gradient). Therefore, we partitioned Sørensen dissimilarity into its turnover and nestedness components to examine the roles of these distinct processes separately. Turnover dominated the total dissimilarity between communities across the BFI gradient compared to nestedness (Fig. S3). For many of the communities analysed, relationships between BFI and nestedness were statistically significant but the effect size and explanatory power of these relationships was small compared to those for turnover-BFI relationships (Fig. S4 and Table S4). This result shows that phylotype replacement drives compositional changes in microbial communities along the BFI gradient.

Turnover of AAV communities was generally higher than for OTU communities across all genes (Fig. S5), indicating a fine-scale partitioning of widespread OTUs into multiple amino acid sequences. Contrastingly, BFI explained substantially more variation in community composition for OTU datasets than for AAVs, presumably reflecting different levels of ecological selection acting on them. Despite occasional differences in the turnover-BFI relationships between AAV and OTU datasets, overall dissimilarity patterns for these datasets were generally strongly correlated, especially for nitrite reducers and methane oxidisers, but less well-correlated for ammonia-oxidising bacteria and methanogens (Table S5).

Stronger patterns at the DNA level (for OTU datasets) than at the amino acid level (AAV datasets) could indicate a higher frequency of synonymous substitutions, leading to higher turnover of DNA sequence diversity relative to amino acid sequence diversity. Thus, environmental selection may not be acting on the physical structure of the enzyme, but could be selecting for distinct strains or species (for an example in AOB, see [45]). Instances where we observed a stronger relationship between BFI and AAV communities (e.g. in the richness of AOA communities) suggest selection on the primary structure of the enzyme that may propagate to/from higher levels of protein structure. Subtle changes in the amino acid sequence of the ammonia monooxygenase enzyme may alter its activity or substrate-affinity [46], with potential biogeochemical implications. Significantly, the active site, or sites, of the archaeal ammonia monooxygenase enzyme remain unresolved [47, 48] and thus variation in the AmoA subunit may have implications for nitrification in geologically contrasting rivers.

### Base flow niches of microbial functional groups

**Fig. 4:**
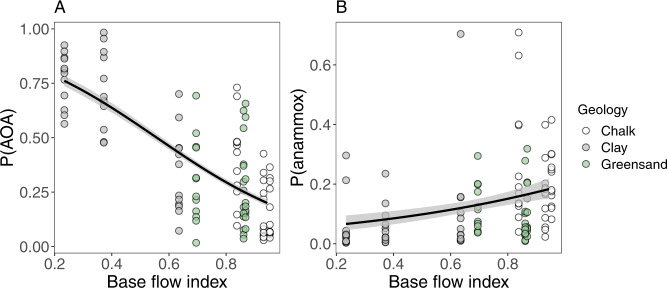
Relationships between base flow index (BFI) and relative abundances of ammonia-oxidisers. Relationships between BFI and the proportion (P) of (**A**) ammonia oxidising archaea (AOA) compared to ammonia oxidising bacteria (AOB), and (**B**) anammox bacteria to aerobic ammonia-oxidisers (sum of AOA and AOB), based on qPCR quantification of the *amoA* (AOA and AOB) and *hzo* (anammox bacteria) genes. Solid lines show the fit of a beta regression model (*P* < 0.0001 in both cases) and grey ribbons are 95% prediction intervals.

Ammonia-oxidising archaea and bacteria, and anammox bacteria potentially compete for the substrate ammonia, and thus we tested for evidence of niche differentiation between these groups in relation to BFI based on gene abundances measured via qPCR. The ratio of AOA *amoA* genes to other ammonia-oxidisers (bacterial *amoA* and anammox *hzo* genes) decreased with increasing BFI (Fig. 4A; coef = 0.03, *z* = −28.24, adj-*D*^*2*^ = 0.56, *P* < 0.001) so that AOA dominated the low BFI clay sediments, and AOB were more abundant in high BFI chalk sediments, reflecting similar observations from permeable Mediterranean catchments [49]. Furthermore, the ratio of anammox bacterial *hzo* genes to aerobic ammonia-oxidiser (AOA and AOB) *amoA* genes increased with BFI (Fig. 4B; coef = 5.06, *z* = *4.48*, adj-*D*^*2*^ = 0.18, *P* < 0.0001), reaching a mean of 21.7% of the total ammonia-oxidising community in the most permeable chalk sediments (BFI = 0.953).

The ratio of bacterial *amoA* and anammox *hzo* gene abundances to bacterial 16S rRNA gene abundance also increased dramatically in high BFI rivers (AOB; coef = 5.47, adj-*D*^*2*^ = 0.23, *P* < 0.0001, hzo; coef = 8.53, adj-*D*^*2*^ = 0.33, *P* < 0.0001) reflecting an increase in the abundance of these groups relative to the total bacterial community as well as archaeal ammonia oxidisers. Bacterial *nirS* gene abundance increased relative to bacterial 16S rRNA gene abundance in high BFI rivers, albeit less markedly than the AOB or anammox bacteria (coef = 2.24, adj-*D*^*2*^ = 0.06, *P* < 0.01), whilst the absolute abundance of bacterial 16S rRNA genes decreased by approximately 32% across the BFI gradient (coef = 0.32, adj-*D*^*2*^ = 0.11, *P* < 0.001).

AOA:AOB ratios are often interpreted as evidence of niche differentiation between these functionally synonymous groups. In the surrounding floodplain soils of the Hampshire Avon, we previously found AOA to be dominant, regardless of underlying geology [19]. In contrast, here we found that ammonia-oxidising communities transition from being AOA-dominated to AOB/anammox-dominated at a BFI of ~0.6, suggesting that catchment permeability impacts fluvial communities but not their counterparts in floodplain soils. AOA usually dominate at low ammonium concentrations due to their greater affinity for ammonium [50], however, the clay sediments in our study catchment had the highest pore-water ammonium concentrations where the AOA dominated (Table S6). Instead, the dominance of AOA in the low BFI sediments may be due to the overriding effects of lower dissolved oxygen concentrations and more acidic pH, both of which favour AOA (Table S6 [19, 51]). The higher relative abundance of anammox bacteria in high BFI gravel sediments compared to low BFI clays suggests the presence of a micro-anoxic niche in these otherwise oxic, permeable sediments, perhaps provided by sediment grain topography and/or stratified biofilm formation [52, 53].

Several of the functional groups showed temporal shifts in their relative proportions between the sampling months (Table S7). However, the importance of accounting for sampling month varied strongly between the different functional groups. The ratio of *nirS* gene copies to total bacterial 16S rRNA gene copies appeared most dependent on sampling month, with inclusion of sampling month improving model fit ~10 fold (Table S7), whereas temporal variation explained little further variance in the ratio of *hzo* gene copies to bacterial 16S rRNA gene copies (Table S7). Temporal variation within our study had negligible impacts on the richness of the functional groups analysed but varying effects on the relative proportions of each functional group. From our study it is not possible to detect whether these effects are seasonal, however if they are, they may be driven by nutrient inputs which have been shown to vary seasonally in these rivers [17]. Over longer temporal scales (years to decades), we would expect the patterns observed here to be relatively stable given that BFI is most strongly related to geology which does not usually vary much over non-geological timescales [15]. However, climate change and land-use shifts in the catchment may invoke more rapid changes in BFI that could in turn impact upon sediment microbiomes over the coming decades [54, 55].

### DNA and protein sequence properties

**Fig. 5:**
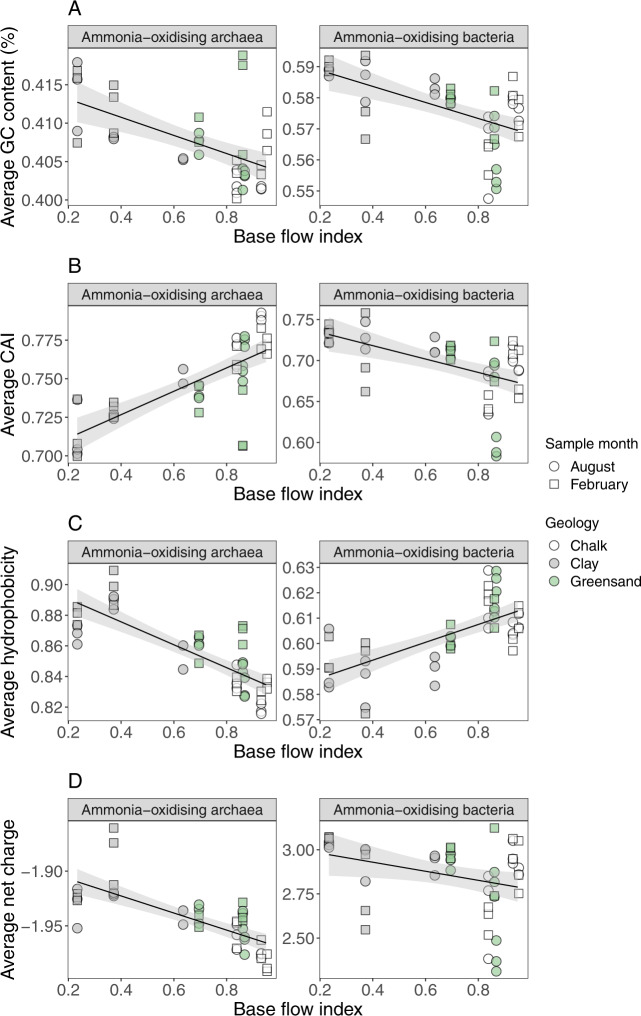
Relationships between base flow index (BFI) and *AmoA* sequence properties. Relationships between river BFI and average (**A**) GC-content of *amoA* genes, (**B**) codon adaptation index, and (**C**) hydrophobicity and (**D**) net charge of AmoA amino acid sequences, for ammonia oxidising archaea (AOA, left) and bacteria (AOB, right). Solid lines indicate fit of a linear regression, with 95% prediction intervals in grey. Average hydrophobicity values were calculated using the Kyte-Doolittle hydrophobicity index and charge was calculated using the Lehninger pKa scale, assuming an intracellular pH of 7. Values were weighted based on the relative abundance of each operational taxonomic unit (**A**, **B**) or amino acid variant (**C**, **D**) in each community.

For both ammonia-oxidising archaea and bacteria, GC-content of *amoA* genes decreased with BFI (Fig. 5A and Table S8), whereas CAI increased in AOA and decreased in AOB (Fig. 5B and Table S8). The shifts in GC-content and CAI of archaeal and bacterial *amoA* genes were accompanied by changes in the average hydrophobicity and net-charge of the translated protein sequences. AOA and AOB showed opposing relationships between BFI and AmoA hydrophobicity (AOA; coef = −0.07, AOB; coef = 0.03, Table S8), with archaeal AmoA protein sequences becoming more hydrophilic in high BFI rivers, and bacterial AmoA sequences becoming more hydrophobic. Despite the contrasting trends of hydrophobicity and CAI between AOA and AOB along the BFI gradient, both groups showed negative relationships between BFI and the average net-charge of their AmoA protein sequence (AOA; coef = −0.08, AOB; coef = −0.25, Table S8). The DNA and protein sequence properties of the other microbial functional genes analysed here were less clearly related to BFI. GC-content and CAI of *nirS* genes showed weak relationships with BFI compared to those of AOA and AOB, whilst both NirS and McrA protein sequences became more negatively charged as BFI increased (NirS; coef = −0.59, *R*^2^ = 0.12, McrA; coef = −1.13, *R*^2^ = 0.15, *P* < 0.05 in both cases, Table S8).

Along the BFI gradient, the sediment changed from silty clays in the low BFI rivers to coarse gravels in the high BFI chalk rivers, likely shifting the dominant mode of life from free-living to biofilm-associated. The observed relationships between BFI and protein sequence properties, particularly for the AmoA sequences, may therefore represent broad physiological responses to the shift in lifestyle between sediment types. The ammonia monooxygenase enzyme is a membrane-bound protein ([56, 57], although see [58] for an example of a soluble form). The charge and hydrophobicity of microbial cell membranes play important roles in biofilm formation [59, 60]. Microbial cells tend to adhere better to surfaces that match their membrane hydrophobicity/hydrophilicity—that is to say that a hydrophobic surface will be more readily adhered to by hydrophobic cells and vice-versa [60]. We found that both AOA and AOB AmoA protein sequences showed opposing hydrophobicity trends along the BFI gradient. Potentially, this suggests that they may adhere to different components of the river sediment that differ in their hydrophobicity, thus spatially differentiating their niche at the scale of individual sediment particles. Ammonia oxidising archaeal and bacterial AmoA protein sequences also showed similar (negative) relationships between net charge and BFI. The more negative charge associated with AmoA proteins in high BFI sites may play a role in acquiring ammonium from the environment [47], although the role of hydrophobicity and charge in substrate affinity and enzymatic function remain unknown.

For AmoA sequences, changes in protein sequence properties along the BFI gradient were accompanied by changes in DNA properties, showing that not only did the identity of amino acids forming these proteins change, but so too did the underlying DNA sequences encoding them. Biased codon use is associated with increased gene expression at the genomic level, but also reflects contrasting lifestyles among microorganisms [61]. Codon usage has also been found to differ between phylogenetic lineages, including in *amoA* sequences from the archaeal orders Nitrosopumilales and Nitrososphaerales [41], both of which were detected by our analysis of archaeal 16S rRNA amplicons. The relationships between CAI and BFI could therefore result from taxonomic turnover across the base flow gradient.

The average GC-content of archaeal and bacterial *amoA* genes also changed with respect to BFI. GC-content may be environmentally driven [62], but is also indicative of growth rate differences between microbial taxa [63]. In AOA, the GC-content of entire *amoA* genes predicts GC-content in the third codon position of each amino acid, potentially explaining concurrent shifts in CAI and GC-content of the archaeal and bacterial *amoA* genes [64]. These coupled relationships may therefore both result from taxonomic turnover along the BFI gradient. Higher GC-content (both across the genome and in the third codon position) has been proposed as an adaptation to oxidative stress in aerobic prokaryotes [65, 66]. However, given that both AOA and AOB showed lower average GC-content in the high BFI river sediments, which had the highest pore water oxygen saturation (Table S6), this explanation seems unlikely.

The *pmoA* gene is evolutionarily related to the *amoA* gene, both being part of the copper-containing membrane-bound monooxygenase family of enzymes [67, 68]. Therefore, it is surprising that we did not observe strong relationships between the sequence properties of *pmoA* genes and BFI, as we did for *amoA* genes. Codon usage biases may arise as the result of limitation of certain resources, such as N [69, 70], and can also improve transcriptional and translational efficiency in certain environments, as observed in type 1a methanotrophs [71]. Consequently, the contrasting relationships observed between the functional groups analysed here may reflect substrate limitations specific to each functional group, as observed in global marine microbiomes [70]. The lack of relationship between BFI and *pmoA* gene properties could alternatively result from a complex interplay between substrate-competition-inhibition effects between ammonia-oxidisers, methanotrophs, and ammonia. Both ammonia- and particulate methane-monooxygenase enzymes can oxidise methane and ammonia [72], but neither ammonia oxidisers or methanotrophs are able to use the energy from the oxidation of the alternate substrate for growth. However, ammonia competitively inhibits the PMO enzyme and produces a toxic product, hydroxylamine, when oxidised. Methanotrophs have different strategies to detoxify hydroxylamine, and these may differ from those of AOB or AOA, thus further setting apart their environmental niche from that of the AOA or AOB based on their ability to deal with reactive N [73].

### Limitations and future directions

An important caveat of our results is that correlation is not causation. BFI is not acting on microbial communities per se—BFI is a synthetic concept, and a microorganism cannot sense the BFI of its habitat afterall. However, BFI is strongly correlated with a number of small-scale variables that do act directly upon microbial communities such as redox profiles, pH, pore water oxygen saturation, and importantly geological sediment type [8, 17, 18, 74]. Furthermore, BFI will not explain all of the variation in microbial community composition and functionality, as river biodiversity is likely structured by a hierarchy of variables acting at different spatial scales. However, as BFI is a large-scale integrative property [16], which constrains the physico-chemical profile of river sediments, it represents a useful proxy for estimating microbial community composition at the regional scale (Fig. S6).

Here, amplicon-based analyses of the target C and N cycling functional groups was required in order to provide sufficient coverage of known rare groups, particularly the anammox bacteria which constitute <1% of the total bacterial community in the sampled river sediments [8], and even less once archaeal and eukaryotic DNA detected by a metagenomic survey is accounted for. However, metagenomic analysis of other membrane-associated enzymes from the wider microbial community, in tandem with analyses of whole-community functional profiles [12], may shed further light on microbial functionalities associated with geological- or hydrological contexts. In particular, metagenomic data may show us the extent to which shifts in microbial lifestyles from geologically distinct rivers constrains the physiological properties of their enzyme encoding protein sequences, and thus their contributions to riverine biogeochemistry.

Establishing the generality of the relationships observed here across a greater diversity of geological settings is a key priority in order to fully disentangle the role of hydrology from geology. In our study sites, sediment types shifted from silty clays to highly permeable coarse gravels along the BFI gradient, reflecting the fact that both of these properties are strongly linked to the underlying geology of a catchment. Therefore, we would expect the relationships observed here between BFI and microbial community properties to be replicable across other catchments. That said, opportunities to unravel potential confounding effects of sediment structure from hydrology may come from sampling rivers with similar base flow conditions but contrasting sediment properties [75]. Alternatively, in situ experiments that manipulate the sediment structure of a river (e.g. to create fish spawning habitat; [76]) would allow us to control for the effects of hydrological variation and biogeographical processes, separating variation solely due to sediment structural changes. Understanding how hydrology and sediment properties interactively shape microbial community structure and functionality will further enhance our ability to make large-scale predictions about the microbial ecology supporting riverine biogeochemistry.

In summary, our results show the statistical power of a single geodiversity variable, catchment permeability, in predicting not only shifts in community composition of C- and N-cycling functional groups, but also genetic and protein properties. Our study highlights the ability of geodiversity variables to overcome what we term the “paradox of scales” that has seen microbial ecologists predominantly focus on the small-scale physico-chemical environment. Questions about the spatial scales at which the environment influences microbial communities remain enigmatic and difficult to answer [77, 78]. In part, this is because we know that microorganisms perceive their environment at microscopic scales [53, 79], and thus even macroecological studies usually consider environmental variables that can vary at small spatial grain [78]. However, because these environmental variables change across very small spatial scales, they do not facilitate a generalised understanding of microbial community ecology that can be extrapolated to larger scales or new sites [80, 81]. Instead, shifting our focus to broader spatial scales by using geodiversity variables as proxies for smaller scale environmental heterogeneity may enable us to make more generalisable links between microbes and their roles in global biogeochemical cycles, a need for which has long been recognised [82, 83]. Overall, our results offer a possible route towards ‘scaling-up’ predictions of fluvial biogeochemistry and explaining differences in the fluxes of C and N between geologically distinct river catchments.

## Supplementary information

Supplementary information
