# Supplementary material for: Hydrological properties predict the composition of microbial communities cycling methane and nitrogen in rivers
Source: ISME Commun. 2022 Jan 21;2:5. doi: 10.1038/s43705-022-00087-7 (PMC9723640; doi:10.1038/s43705-022-00087-7)
Supplement: Supplementary file 1 — Supplementary information [file 43705_2022_87_MOESM1_ESM.pdf]

Supplementary Information

Table S1. Details of phylogenetic and functional genes targeted in qPCR and amplicon sequencing. Primer sequences follow IUPAC nucleotide codes.

Gene	Primer	Primer sequence (5′-3′)	Reference
Archaeal 16S rRNA	344F<br>915R	ACGGGGYGCAGCAGGCGCGA<br>GTGCTCCCCCGCCAATTCCT	Raskin *et al.*, 1994<br>Stahl & Amann, 1991
Bacterial 16S rRNA	S-D-Bact-0341-b-S-17<br>S-D-Bact-0785-a-A-21	CCTACGGGNGGCWGCAG<br>GACTACHVGGGTATCTAATCC	Klindworth *et al.*, 2013
Archaeal *amoA*	CrenAmoA-23F<br>CrenAmoA-616R	ATGGTCTGGCTWAGACG<br>GCCATCCATCTGTATGTCCA	Tourna *et al.*, 2008
Bacterial *amoA*	AmoA-1F<br>AmoA-2R	GGGGTTTCTACTGGTGGT<br>CCCCTCKGSAAAGCCTTCTTC	Rotthauwe *et al.*, 1997
hzo	HZO-1F<br>HZO-1R	AAGACNTGYCAYTGGGGWAAA<br>GACATACCCATACTKGTRTANACNGT	Long *et al.,* 2013
nirS	cd3aF<br>R3cd	AACGYSAAGGARACSGG<br>GASTTCGGRTGSGTCTTSAYGAA	Throbäck *et al.*, 2004
mcrA	mlas<br>mcrA-rev	GGTGGTGTMGGDTTCACMCARTA<br>CGTTCATBGCGTAGTTVGGRTAGT	Steinberg and Regan, 2009
pmoA	A189-F<br>A650-R	GGNGACTGGGACTTCTGG<br>ACGTCCTTACCGAAGGT	Holmes *et al.*, 1995<br>Bourne *et al.*, 2001
For Illumina MiSeq sequencing, each locus-specific primer set was modified to include Illumina-specific overhang sequences as follows:<br>TCGTCGGCAGCGTCAGATGTGTATAAGAGACAG-(Forward primer 5’-3’)<br>GTCTCGTGGGCTCGGAGATGTGTATAAGAGACAG-(Reverse primer 5’-3’)			

Table S2. The number of sequences per sample and total richness after removal of singleton operational taxonomic units (OTUs) or Amino Acid Variants (AAVs) and rarefaction for each gene and dataset (AAV and OTU). Samples with fewer sequences were removed prior to AAV/OTU clustering.

Gene dataset	Community type	Number of sequences per sample	Total sample size[a]	Total richness
Archaeal 16S rRNA	OTU	636	47	537
Bacterial 16S rRNA	OTU	3491	48	18327
AOA *AmoA*	AAV	1743	42	870
	OTU	1803		102
AOB *AmoA*	AAV	2586	45	7428
	OTU	2626		118

nirS	AAV	3244	45	11746
	OTU	3832		4983
hzo	AAV	16661	45	7475
	OTU	16981		285
mcrA	AAV	4793	43	11608
	OTU	4951		1328
pmoA	AAV	3454	45	9787
	OTU	3606		143

[a] Final sample sizes were quantified after quality filtering and rarefaction of data. Discarded samples therefore include those that were lost during the QC process or were discarded following manual inspection of library sizes.

Table S3. Results of likelihood ratio tests comparing models of AAV or OTU richness as a function of base flow index with and without sample month interactions. For most genes, temporal variation in the richness of AAVs or OTUs was not statistically significant. Significant P values are highlighted with bold text.

Gene dataset	Community type	Model 1 Richness ~ bfi			Model 2 Richness ~ bfi * month			LR statistic	*P* value
		AIC	Adj-D^2	Residual df	AIC	Adj-D^2	Residual df		
Archaeal 16S rRNA	OTU	439.3	0.12	42	442.4	0.09	40	0.94	0.63
Bacterial 16S rRNA	OTU	666.4	0.01	46	668.5	0.01	44	1.95	0.38
Archaeal *amoA*	AAV	392.2	0.25	40	386.1	0.37	38	10.04	**** < 0.01**
	OTU	274.5	-0.03 (0.00)	40	276.4	-0.03 (0.00)	38	2.02	0.36
Bacterial *amoA*	AAV	563.4	0.10	43	563.4	0.14	41	3.99	0.14
	OTU	328.6	0.35	43	329.7	0.36	41	2.87	0.24
hzo	AAV	574.2	0.12	43	578.1	0.07	41	0.14	0.93

	OTU	437.7	0.20	43	440.2	0.18	41	1.50	0.47
nirS	AAV	589.0	-0.04 (0.00)	43	590.2	-0.03 (0.00)	41	2.73	0.26
	OTU	586.3	-0.05 (0.00)	43	587.9	-0.04 (0.00)	41	2.49	0.29
mcrA	AAV	634.3	-0.01 (0.00)	41	634.9	0.02	39	3.36	0.19
	OTU	556.0	0.01	41	556.7	0.02	39	3.21	0.20
pmoA	AAV	593.9	-0.03 (0.00)	43	597.0	-0.06 (0.00)	41	0.87	0.65
	OTU	345.6	0.00	43	343.3	0.10	41	6.27	* **0.04**

Table S4. Results of negative exponential GLMs between pairwise differences in base flow index and either the nestedness or turnover components of microbial β-diversity, for all genes and community types (operational taxonomic unit (OTU) or amino acid variant (AAV)).

Gene	Community type	Nestedness			Turnover		
		Coefficient	R^2	*P* value	Coefficient	R^2	*P* value
Archaeal 16S rRNA	OTU	-0.02	0.00	0.28	0.24	0.04	0.001
Bacterial 16S rRNA	OTU	0.00	0.00	0.121	0.31	0.08	0.001
AOA *amoA*	AAV	-0.02	0.00	0.04	0.83	0.33	0.001
	OTU	-0.05	0.02	0.001	0.80	0.52	0.001
AOB *amoA*	AAV	-0.03	0.03	0.001	0.91	0.24	0.001
	OTU	0.13	0.03	0.001	0.16	0.04	0.001
hzo	AAV	0.02	0.01	0.001	0.24	0.16	0.001
	OTU	0.11	0.05	0.001	0.05	0.01	0.001
nirS	AAV	-0.02	0.03	0.001	1.01	0.55	0.001
	OTU	-0.03	0.03	0.001	1.03	0.50	0.001

mcrA	AAV	-0.06	0.05	0.001	0.65	0.27	0.001
	OTU	-0.13	0.02	0.001	0.44	0.19	0.001
pmoA	AAV	-0.02	0.01	0.001	0.31	0.10	0.001
	OTU	-0.12	0.02	0.001	0.68	0.23	0.001

Table S5. Correlations between the community dissimilarity of AAV and OTU datasets for each functional group, determined by Mantel tests with 1000 permutations.

Functional gene	Mantel test statistic	P value
Archaeal amoA	0.69	< 0.001
Bacterial amoA	0.49	< 0.001
hzo	0.63	< 0.001
nirS	0.97	< 0.001
mcrA	0.47	< 0.001
pmoA	0.96	< 0.001

Table S6. Summary of porewater chemistry characteristics. Measurements were conducted at sediment depths <5cm, as described by Lansdown *et al*. (2016).

Site	pH	O_2 saturation (% of surface water)	Nitrite (μM)	Nitrate (μM)	Ammonium (μM)
Clay 1	7.17 ± .04	15.41 ± 3.60	0.98 ± 0.14	20.35 ± 5.58	171.87 ± 30.85
Clay 2	7.00 ± 0.05	6.09 ± 1.02	1.48 ± 0.37	18.74 ± 10.78	207.96 ± 31.22
Clay 3 *	-	-	-	-	-
Greensand 1	7.58 ± 0.04	53.58 ± 6.66	0.59 ± 0.15	172.17 ± 27.68	15.57 ± 4.57
Greensand 2	7.42 ± 0.03	24.44 ± 4.06	0.65 ± 0.24	66.22 ± 22.12	15.78 ± 4.86
Greensand 3 *	-	-	-	-	-
Chalk 1	7.80 ± 0.04	49.64 ± 8.50	2.77 ± 0.5	456.76 ± 24.18	5.56 ± 1.67
Chalk 2	7.83 ± 0.04	64.43 ± 5.19	1.16 ± 0.2	387.82 ± 18.74	5.05 ± 1.23

Chalk 3 *	-	-	-	-	-
* Sites Clay 3, Greensand 3 and Chalk 3 were visited only to collect sediment samples for the molecular analyses presented, and were not subject to detailed porewater analyses as described in Lansdown *et al*., (2016).					

Table S7. Results of likelihood ratio tests comparing models of the proportional abundance of different functional groups as a function of base flow index with and without sample month interactions.

Ratio	Model 1 Ratio ~ bfi			Model 2 Ratio ~ bfi * month			Test statistic	*P* value
	AIC	Adj-D^2	Residual df	AIC	Adj-D^2	Residual df		
AOA amoA:total ammonia oxidisers	-86.9	0.56	103	-148.8	0.79	97	73.96	< 0.001
hzo:total ammonia oxidisers	-223.3	0.18	103	-230.5	0.29	97	19.17	< 0.01
AOB amoA:Bacterial 16S rRNA	-970.1	0.23	103	-1014.5	0.58	97	56.40	< 0.001
nirS:Bacterial 16S rRNA	-408.1	0.06	103	-511.5	0.70	97	115.47	< 0.001
hzo:Bacterial 16S rRNA	-223.3	0.18	103	-230.5	0.29	97	2.89	0.82

Table S8. Results of linear models analysing the relationship between BFI and DNA sequence (GC-content, codon adaptation index) or amino acid sequence (hydrophobicity, net charge) properties for each of the functional genes analysed. All metrics were calculated for each community, and weighted by the relative abundance of each OTU (for DNA properties) or AAV (for amino acid properties).

Sequence variable	Statistic	AOA *AmoA*	AOB *AmoA*	*hzo*	*nirS*	*mcrA*	*pmoA*
GC-cont	Coefficient	-0.01	-0.03	-0.01	0.01	0.00	0.00

ent	Test statistic	-4.42	-4.60	-3.12	4.89	-0.01	-1.09
	R^2	0.31	0.31	0.17	0.24	0 (-0.02)	0.00
	P value	<0.001	<0.001	<0.01	<0.001	0.99	0.28
Codon adaptation index	Coefficient	0.08	-0.08	-0.01	0.04	-0.02	-0.01
	Test statistic	7.07	-4.00	-0.62	4.23	-1.92	-0.69
	R^2	0.54	0.25	0 (-0.01)	0.28	0.06	0 (-0.01)
	P value	<0.001	<0.001	0.54	<0.001	0.06	0.50
Hydrophobicity	Coefficient	-0.07	0.03	0.01	-0.01	0.03	-0.02
	Test statistic	-8.47	6.07	1.52	-0.80	2.90	-1.54
	R^2	0.63	0.45	0.03	0 (-0.01)	0.15	0.03
	P value	<0.001	<0.001	0.14	0.43	<0.01	0.13
Net charge	Coefficient	-0.08	-0.25	0.02	-0.59	-1.13	-0.01
	Test statistic	-6.84	-2.17	0.36	-2.61	-2.90	-0.30
	R^2	0.53	0.08	0 (-0.02)	0.12	0.15	0 (-0.02)
	P value	<0.001	<0.05	0.72	<0.05	<0.01	0.77

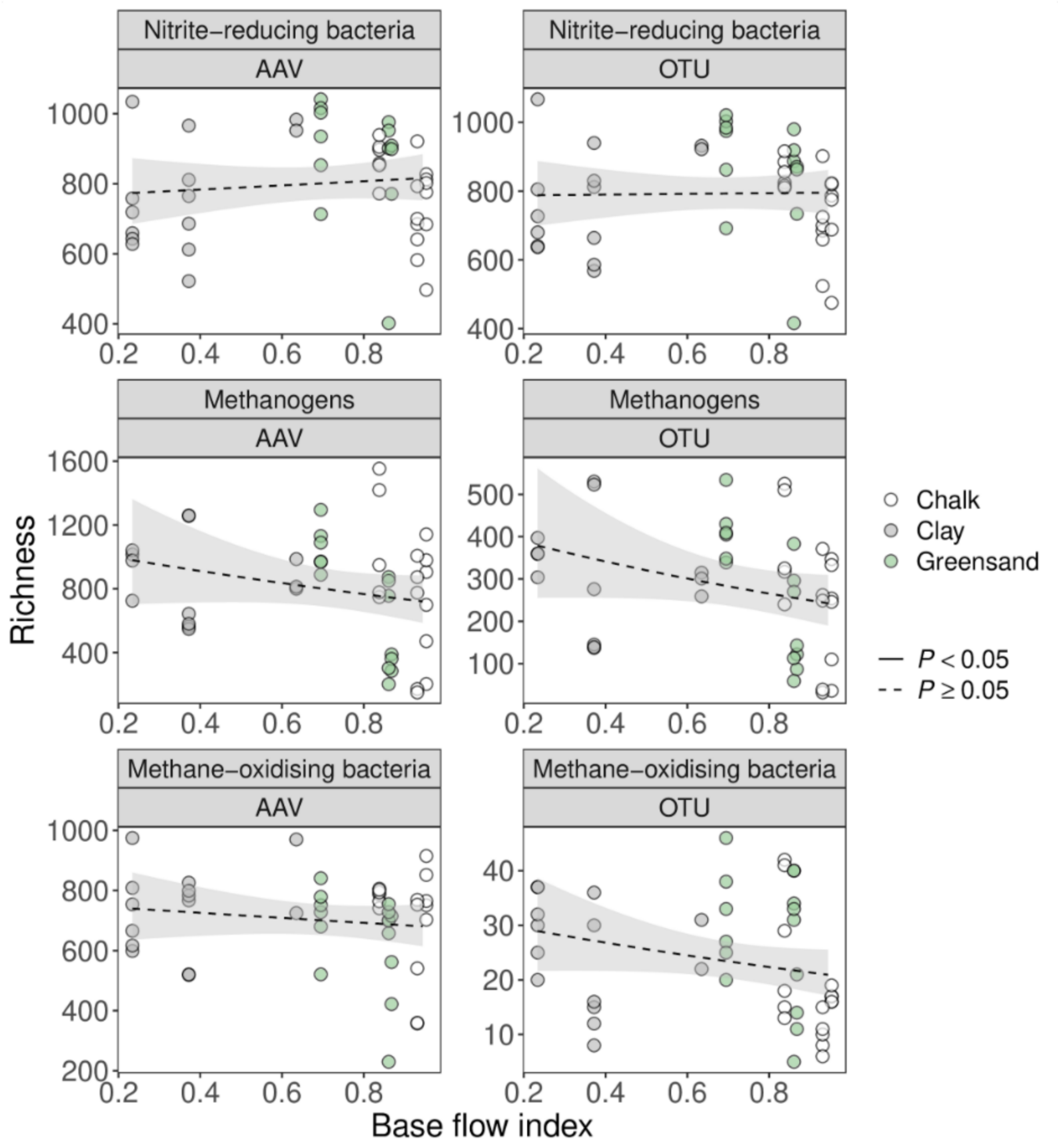

Figure S1. Relationships between base flow index (BFI) and richness of amino acid variant and operational taxonomic unit based communities for (A) nitrite reducing communities (*nirS*), (B) methanogen communities (*mcrA*), and (C) methanotrophic communities (*pmoA*).

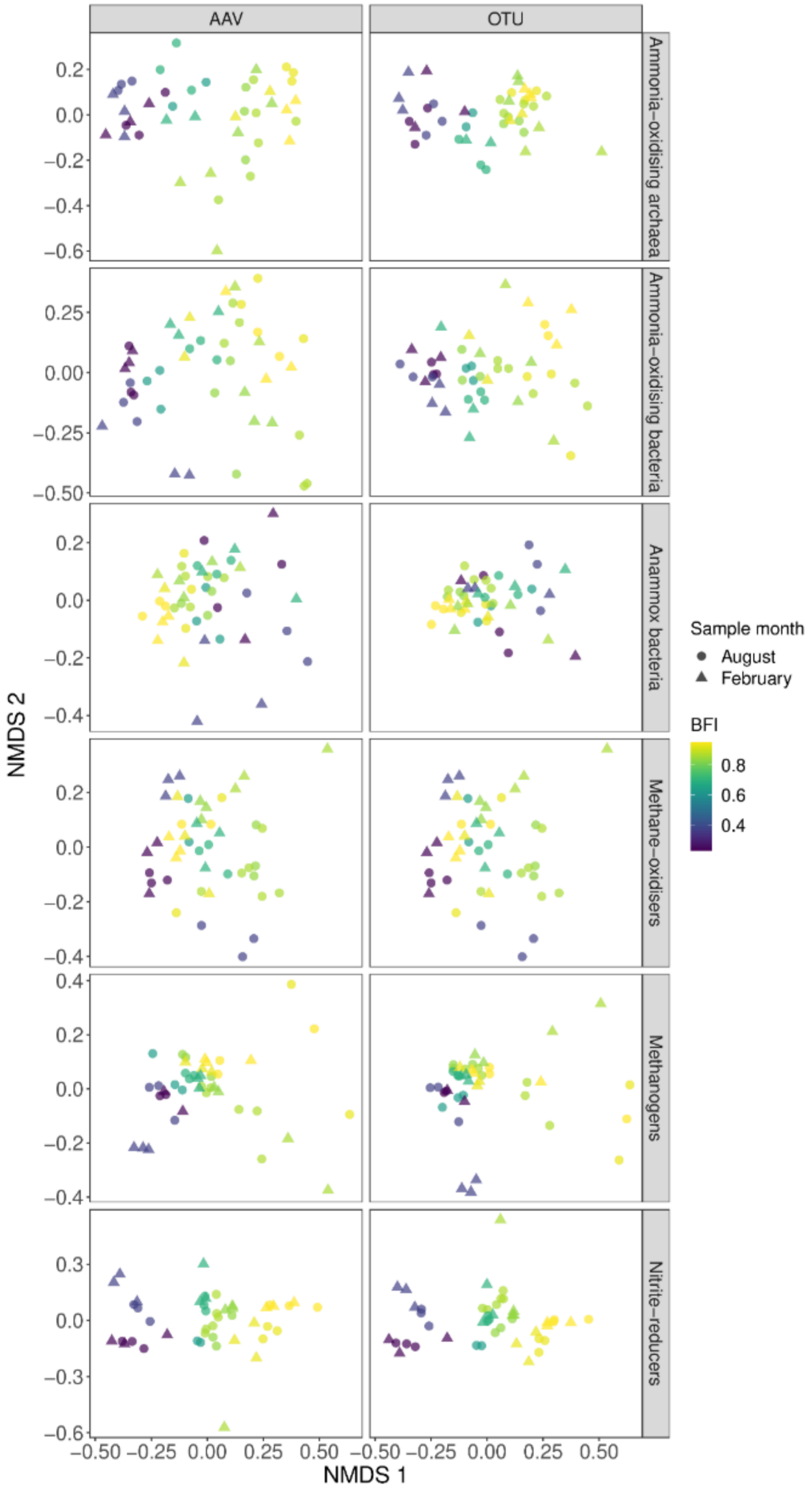

Figure S2. Non metric multidimensional scaling (NMDS) plots for each functional group, based on both amino acid variant (AAV) and operational taxonomic unit (OTU) datasets. NMDS analyses were based on Sorensen dissimilarity matrices. Scale shows the base flow index (BFI) for the river each community was sampled from. Points closer together indicate communities that are compositionally similar.

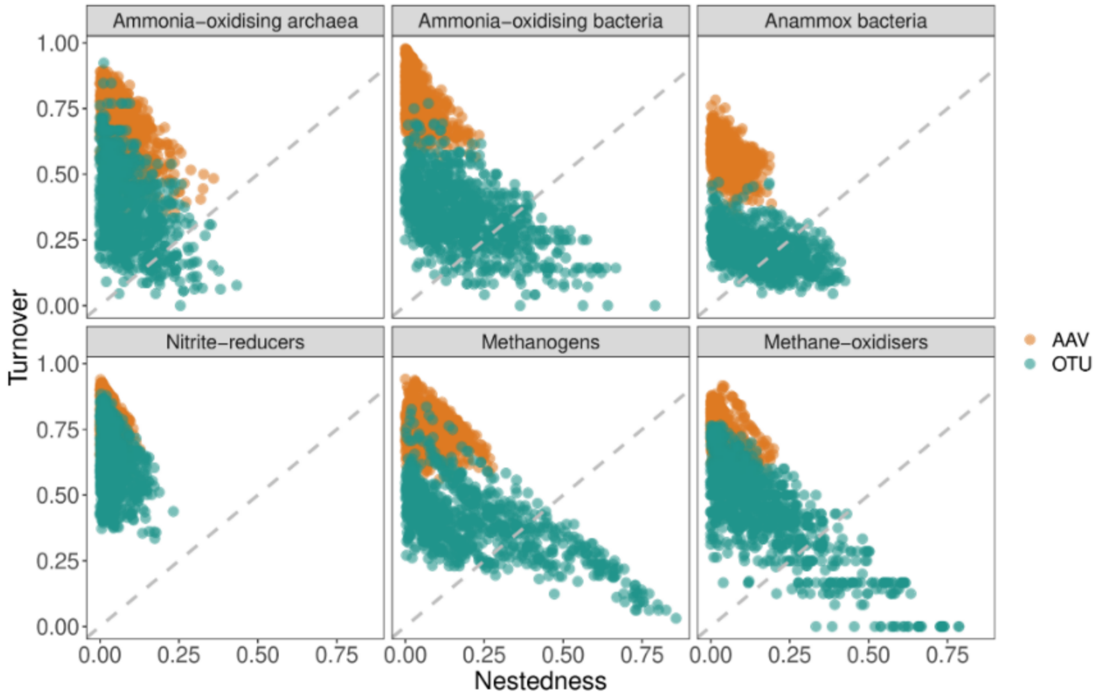

Figure S3. Comparison of nestedness (the subsetting of communities along the base flow gradient) and turnover (the replacement of species along the base flow gradient) components of β-diversity for AAV- and OTU-based communities for each functional group. Points that lie to the left of the dashed line indicate that turnover was dominant over nestedness, whereas points to the right show comparisons where nestedness dominated over turnover.

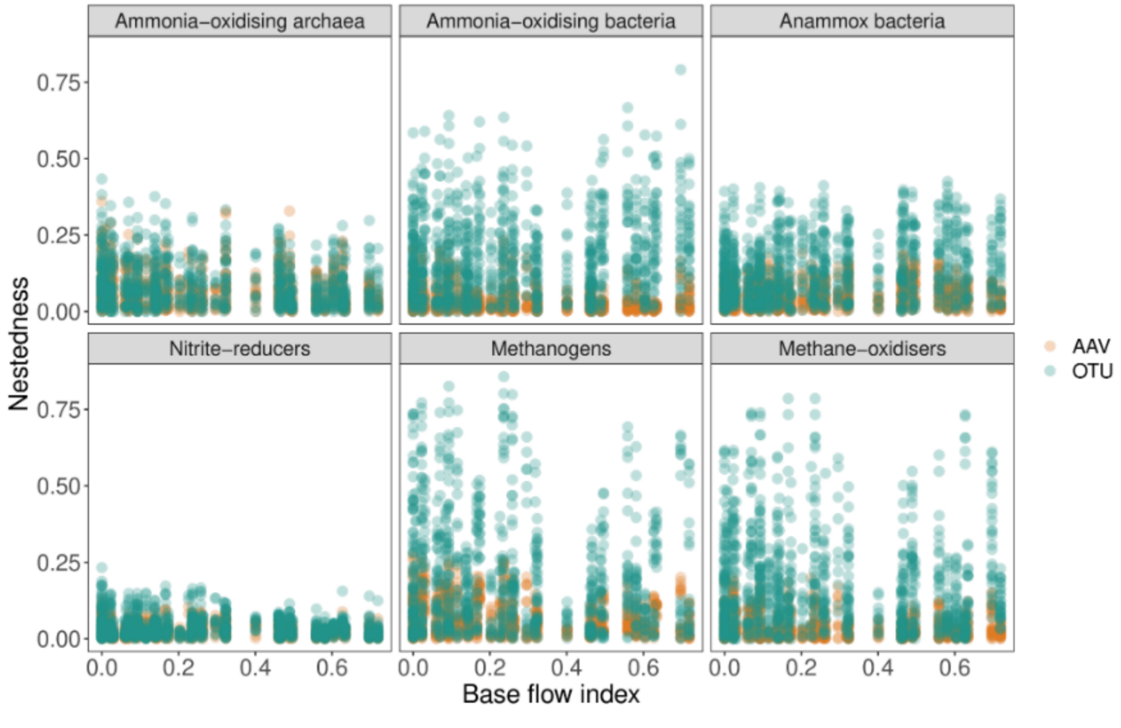

Figure S4. The relationship between the pairwise nestedness component of β-diversity and the difference in river base flow index (BFI) for all functional groups and community types.

GLMs showed no statistically significant relationship between BFI and nestedness for any functional groups.

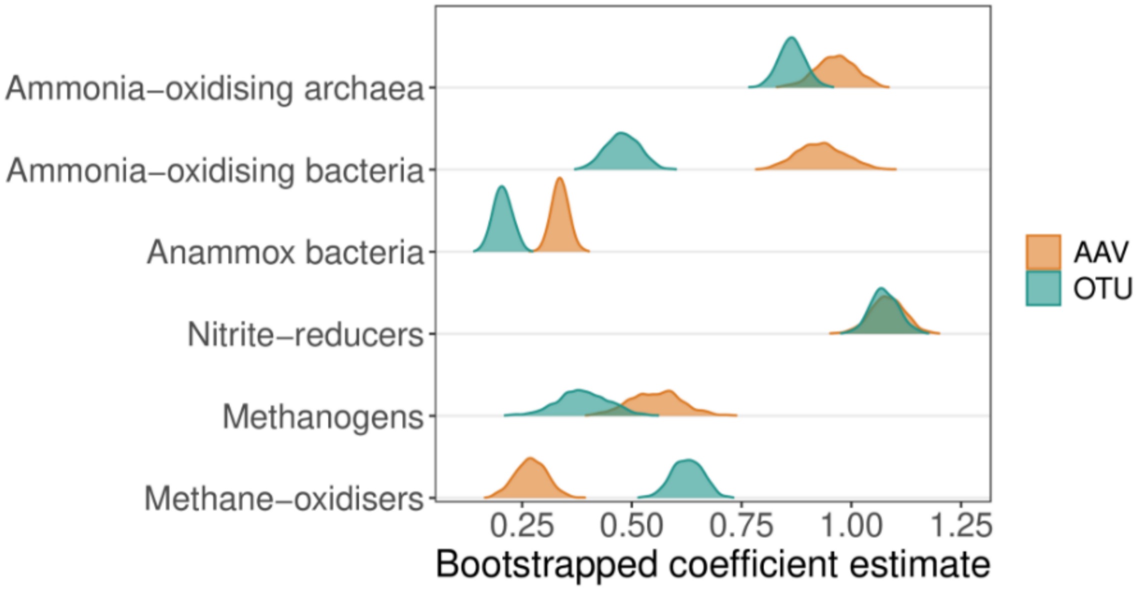

Figure S5. Bootstrapped coefficient estimates of negative exponential models fitted to relationships between pairwise differences in base flow index (BFI) and the turnover component of β-diversity. Distributions are based on 1000 bootstraps.

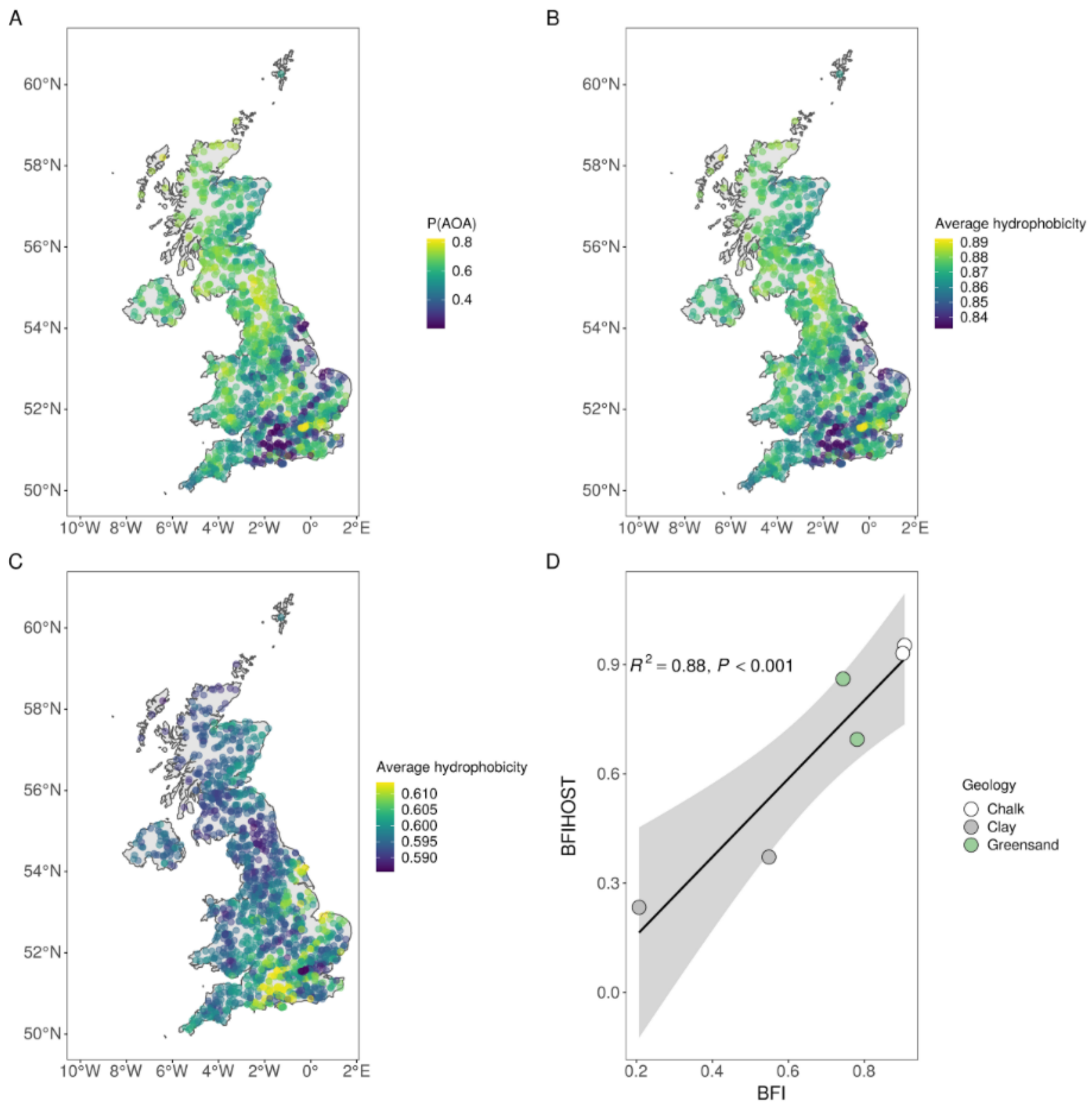

Figure S6. Model predictions of the (A) AOA:AOB ratio and average hydrophobicity of archaeal (B) and bacterial (C) *AmoA* amino acid sequences. Predictions are made using the base flow index based on the hydrology of soil types (BFIHOST) classification described by (Griffin *et al.*, 2019), and downloaded from the UK National River Flow Archive with the “rnrfa” R package (Vitolo *et al.*, 2016). For rivers within the Hampshire-Avon catchment, the BFIHOST metrics are highly similar to those previously reported for a subset of the rivers sampled here (panel D, Heppell *et al.*, 2017).
